# A Comparison Between the Expansion Force Exerted by Thermo-Printed Aligners and 3D Printed Aligners: An In Vitro Study

**DOI:** 10.3390/bioengineering12090912

**Published:** 2025-08-25

**Authors:** Samuele Avolese, Simone Parrini, Andrea Tancredi Lugas, Cristina Bignardi, Mara Terzini, Valentina Cantù, Tommaso Castroflorio, Emanuele Grifalconi, Nicola Scotti, Fabrizio Sanna

**Affiliations:** 1 Department of Mechanical and Aerospace Engineering, Polytechnic University of Turin, 10100 Torino, Italy; 2 Specialization School in Orthodontics, Department of Surgical Sciences, Dental School of the University of Torino, 10100 Torino, Italy; 3 PolitoBIOMed Lab, Polytechnic University of Turin, 10100 Torino, Italy; 4 Independent Researcher, 10100 Torino, Italy; 5 Department of Surgical Sciences, Dental School of the University of Torino, 10100 Torino, Italy

**Keywords:** 3D printed aligners, orthodontic expansion, aligner biomechanics, force measurement, mixed dentition, digital orthodontics

## Abstract

Background: The fabrication of orthodontic aligners directly via three-dimensional (3D) printing presents potential to increase the efficiency of aligner production relative to traditional workflows; however, several aspects of the 3D printing process might affect the dimensional fidelity of the fabricated appliances. The aim of this study is to measure the forces expressed by a 3D printed aligner made with TC-85 DAC resin (Grapy Inc., Seoul, Republic of Korea) when an expansion movement of the entire upper dental arch is programmed, comparing the measured forces with those obtained by a common thermoformed aligner (Smart Track^®^, Align Technology, Santa Clara, CA, USA). Materials and methods: A patient in transitional mixed dentition was selected, with the presence of all the first molars and permanent upper and lower incisors, and the canines and premolars have not started the exchange. From this patient, a virtual set up of the upper arch has been planned with an expansion of 0.2 mm and 0.4 mm per side; 3 mm horizontal rectangular attachments were added to the set up on the vestibular surface of the permanent molars, deciduous premolars, and deciduous canines. On this set up, 10 Smart Track aligners and 10 3D printed aligners with TC-85 DAC resin were produced. The fabricated aligners were mounted on the machinery used for the test (ElectroForce^®^ Test Bench; TA Instruments, New Castle, DE, USA) by means of specific supports that simulate the upper arch of the patient (divided into two sides: right and left). To simulate the intraoral environment, the measurements were carried out in a thermostatic bath at a temperature of 37 °C. Results: The key results of this paper showed differences between Smart Track^®^ and TC-85 DAC. In particular, the expanding force exerted by the 0.2 mm per side expanded Smart Track^®^ aligners was on average +0.2162 N with a D.S. of ±0.0051 N during the 8 h; meanwhile, the force exerted by the 0.2 mm per side expanded TC-85 DAC 3D printed aligners was on average −0.0034 N with a D.S. of ±0.0036 N during the 8 h. The force exerted by the 0.4 mm per side expanded Smart Track^®^ aligners was on average +0.7159 N with a D.S. of ±0.0543 N during the 8 h; meanwhile, the force exerted by the 0.4 mm per side expanded TC-85 DAC 3D printed aligners was on average +0.0141 N with a D.S. of ±0.004 N during the 8 h. Conclusions: Smart Track^®^ aligners express a quantitatively measurable force in Newtons during the programmed movements to obtain a posterior expansion of the dental arches; on the contrary, aligners made with TC-85 DAC resin, in light of the results obtained from this study, express forces close to 0 during the realization of the movements programmed to obtain a posterior expansion of the dental arches.

## 1. Introduction

In recent decades, the advancement of digital technologies has brought great changes and improvements to all medical fields, including dentistry. In the orthodontic field, the biggest clinical and technological innovation of digital progress has been the introduction of clear aligner therapy.

The performances of clear aligners are improved year after year thanks to the development of new materials and the constant search for improvements in their properties.

Clear aligners are produced for the most part, conventionally, through the “vacuum” thermoforming method with transparent, biocompatible thermoplastic materials, carried out directly on a pre-made dental model [1].

Thermoforming is a production process during which geometric inaccuracies can be induced that affect the orthodontic forces expressed and the fitting of the clear aligners on the dentition, making the tooth movements less predictable [2].

To overcome the limitations of the conventional manufacturing method, the direct 3D printing of clear aligners has recently been introduced. This method takes less time and should also result in fewer geometric inaccuracies [2,3].

While the application of 3D printing for orthodontic aligners is one of the most dynamic areas of innovation, additive manufacturing is also being increasingly adopted for the fabrication of permanent intraoral restorations, such as crowns, bridges, and prosthetic frameworks. These developments demonstrate the growing relevance of 3D printing in broader areas of dentistry. Balestra et al. recently reviewed the literature on 3D printed materials used for definitive restorations, highlighting both the potential of this technology and the current limitations due to scarce and methodologically heterogeneous evidence [4]. They underline the need for standardized research protocols to support long-term clinical use.

A 3D printable and biocompatible material approved by the Korea Food and Drug Administration (KFDA) and the European Commission (EC) has been developed. Infrared spectroscopy analysis indicated the presence of an aliphatic polymer vinylester–urethane, probably functionalized methacrylate [5,6].

According to the authors’ knowledge, scientific studies still lack clinical and technical indications on these materials. Furthermore, according to the existing literature, there is no evidence of comparison of the force exerted between Smart Track^®^ (Align Technology, Santa Clara, CA, USA) and TC-85 DAC resin (Grapy Inc., Seoul, Republic of Korea) aligners, which are widely used in contemporary orthodontics.

The primary purpose of this study is to measure and compare the magnitude of expansion force expressed by an aligner printed with 3D printing technology using TC-85 DAC resin and that expressed by a Smart Track^®^ thermoformed aligner.

The null hypothesis is that the forces exerted by the two types of clear aligners taken into consideration are the same during the expansion of the upper arch in a mixed dentition patient.

The aim of the study is to measure and compare the magnitude of force exerted during a programmed expansion of 0.2 mm and 0.4 mm per side by directly 3D printed aligners with TC-85 DAC resin and Smart Track^®^ aligners made with a thermoforming technique.

## 2. Materials and Methods

Using a digital intraoral scan of a patient in transitional mixed dentition, 10 3D printed aligners with TC-85 DAC resin and 10 thermoformed aligners manufactured by Smart Track^®^ were made, programming expansion movements of 0.2 mm and 0.4 mm per side in the treatment plan. The 0.4 mm activation aligner was not a real clinical protocol activation but is useful for the purpose of this in vitro study.

The total number of samples analyzed was therefore divided as follows:

A total of 20 thermoformed aligners manufactured by Smart Track^®^ (10 with a 0.2 mm expansion per side and 10 with a 0.4 mm expansion per side).

A total of 20 3D printed aligners using TC-85 DAC resin (10 with a 0.2 mm expansion per side and 10 with a 0.4 mm expansion per side).

The study was approved by the local ethics committee (00027/2022).

### 2.1. Patient Selection

A patient in transitional mixed dentition was selected, with only the first molars present in the arch, permanent upper and lower incisors, and a state of vertebral maturation classifiable CVMS 3, according to the classification of Franchi and Baccetti [7].

The intraoral scan was detected using iTero Element 2 scanners (© 2020 Align Technology, Inc.), selecting only the digital model of the upper arch, exported in STL format using “OrthoCAD 5.9.1.50” software.

### 2.2. Conception of Orthodontic Set Up

Starting from the STL file, virtual set ups were created that included an expansion of 0.2 mm and 0.4 mm per side. The 3 mm horizontal rectangular attachments were added to the set up on the vestibular surface of the permanent molars, molars, and canines in the deciduous dentition in order to ensure correct anchorage during transverse expansion movements.

This virtual set up was used for all the samples in the study.

### 2.3. Realization of Tester Aligners

For Smart Track^®^ aligners, Clin Check software (Align Technology, Santa Clara, CA, USA) was used to achieve the configuration described above.

Smart Track^®^ aligners are manufactured by Align Technology using a thermoforming process, with a material consisting of a multilayer aromatic thermoplastic polyurethane/co-polyester and with the manufacturer’s stated thickness of 0.75 mm (0.030″) [8,9].

The aligners printed with 3D technology were designed using Direct-Aligner uDesign beta software, respecting the set up described above and programmed with a thickness of 0.75 mm (in order to have the same thickness as Invisalign aligners) and an off-set of 0.05 mm, creating with the same software the supports necessary for printing. In addition, a posterior transversal support useful for stabilizing the programmed intermolar distance was created.

These devices were printed using a Sprint Ray Pro95 printer at 100 μm thickness per layer. For the pre-curing of the TC-85 DAC resin used, the printer was set to the “SprintRay US—SprintRay Splint” program. At the end of this phase, the excess resin was removed through 2 centrifuge cycles 6 min each and the print supports were manually eliminated by means of digital pressure; finally, cotton pellets were used to remove excess residual resin on the inside of the attachments. Then, the aligners thus produced were cured in a nitrogen-saturated environment [10,11] for 14 min by a Cure M machine (Graphy Inc., Seoul, Republic of Korea), as indicated by the manufacturer. Finally, the posterior cross-support was removed using rotating instruments.

### 2.4. Model Test Creation

The STL of the original T0 intraoral scan (equipped with the programmed attachments) was digitally hoofed and digitally sectioned along the median sagittal plane into two halves, one right and one left, leaving a gap of 1 mm on each side from the median sagittal plane.

The incisors were removed from the original STL file, as they could have altered the forces expressed by the aligner in the posterior sectors. On the external sides (vestibular-lateral) of the two halves, the supports (one for the right half and one for the left half) necessary for future housing in the machine to take the measurements were digitally inserted. The two halves thus designed were 3D printed using a resin from models (SprintRay Die and Model 2 gray) in order to have a physical support on which the aligners can express the expansive force to the programmed extent. The housing of the two halves of the model in the machine used for force detection was made possible thanks to the creation (again by 3D printing) of a suitable positioning support.

### 2.5. Practical Measurement of Expansion Forces

The two physical halves of the T0 model were then mounted using a template to ensure repeatable positioning on the machine used for the test, which was the ElectroForce^®^ Planar Biaxial Test Bench; TA Instruments, New Castle, DE, USA. The test protocol was set up so that the motors of the testing machine kept the 3D model in the starting position in order to measure the expansion force expressed by the devices on it by means of a 22 N load cell.

To simulate the intraoral environment, measurements were taken in a thermostatic bath at a temperature of 37 °C. This is necessary because the TC-85 DAC resin ideally expresses the programmed force at this temperature, as this material has shape memory.

In addition, to make it easier to insert the 3D printed aligner, the manufacturer suggests preheating the aligner to <60 °C. Each aligner of the two TC-85 DAC resin groups was then immersed in a thermostatic bath at 65 °C for 2 min before insertion on the test model.

**Figure 1:**
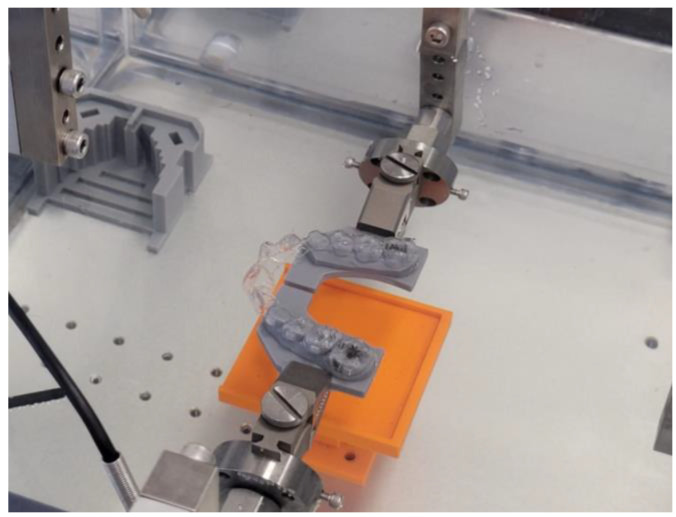
Custom mechanical testing device used to evaluate the forces expressed by a clear aligner on a 3D-printed dental model.

The Smart Track^®^ aligners were instead inserted directly on the test model (Figure 1).

Once the aligners were inserted into the two halves of the test model mounted on the testing machine, they were left for 8 consecutive hours each, during which the testing machine measured the force exerted over time by each aligner inserted.

The values obtained for the amount of force exerted by the various sets of aligners tested are expressed in Newtons; positive values indicate an expanding force exerted, information programmed in the virtual set ups, while negative values indicate a force with a direction of contraction (opposite to the programmed one).

The forces were recorded in real time, with sampling carried out at 1 s time intervals, obtaining 28,800 values distributed over 8 h.

## 3. Statistical Analysis and Results

SPSS software (IBM Corporation, 1 New Orchard Road, Armonk, NY, USA) was used for statistical analysis.

Power analysis based on the observed effect sizes and sample size (n = 10 per group) indicated adequate statistical power for the main comparisons. For instance, the contrast between SmartTrack^®^ and TC-85 DAC aligners with the 0.2 mm expansion showed a large effect size (Cohen’s d = 1.54) and a power of approximately 90% (α = 0.05), confirming the reliability of the findings.

The first 90 s were removed from each signal (corresponding to a tested aligner) (in order to remove an equal time from all tests that would allow for the elimination of the assembly phase of the device) and the measurement was considered until the end of the 8 h. This way, the signals are all the same length and a more reliable comparison is possible.

**Figure 2:**
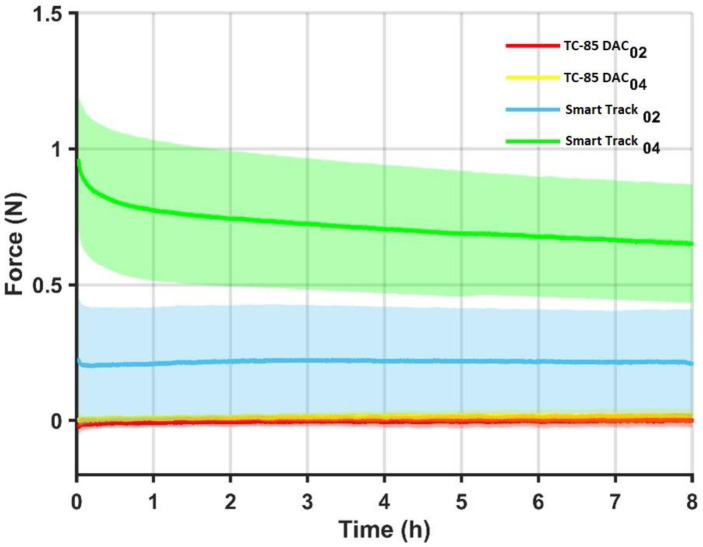
Force decay over time for TC-85 DAC and SmartTrack^®^ aligners at 0.2 mm and 0.4 mm programmed displacements.

For the 10 curves of each device, the mean and standard deviation of the force trend over time were calculated, represented in the figure below (Figure 2), in which the solid lines represent the average curve for each device and the halos around them the dispersion of the data (mean + st.dev and average − st.dev).

The average force values expressed over 8 h by each group of 10 aligners, with the relative standard deviations (indices of the variability of the individual aligners in each group of 10) summarized in the table below (Table 1). Together with these values, the field of variation in the average of each group was added over the course of 8 h. This value is an index of the variability over time of the average of each group of aligners.

Finally, a comparison was made using two-tailed T-tests (5% significance level) between the forces exerted by the four groups examined: it emerges that there is statistically significant variability both between the two groups of 3D printed aligners with TC-85 DAC resin and between the groups of Smart Track aligners when compared to those with TC-85 DAC resin and between the two groups of Smart Track; all with *p* < 0.0001.

## 4. Discussion

In recent decades, clear aligners have seen an increase in use and proposal in orthodontic patients; in fact, compared to traditional fixed braces, clear aligners seem to have some advantages, including not imposing dietary restrictions and better esthetics [12]. 

Recently, the attention of scientific research in the orthodontic field, particularly regarding clear aligners, has focused on the study of materials and means to make aligners as effective and efficient as possible in one’s own practice.

This trend reflects a broader movement in biomedical engineering, where additive manufacturing technologies are increasingly applied to produce patient-specific medical devices with optimized properties, as extensively discussed in the recent literature on 3D printed metallic biomaterials [13].

Additive manufacturing has, in fact, experienced exponential growth across multiple fields, including biomedical applications. A recent bibliometric analysis identified nearly 80,000 scientific documents on AM indexed in Scopus as of February 2025, with a significant rise in publications from 2022 to 2024, particularly in engineering and materials science [14].

In the orthodontic field, the increasing diffusion of the practice of producing aligners directly "in office" by the orthodontist himself has attracted the attention of an increasing number of professionals who have begun to intensify the search for new materials and resins compatible with 3D printing techniques, which could meet the need for a demand for greater precision, a shortening of the supply chain, and a reduction in costs (not having to produce physical models) [2,15].

The shift toward the in-office fabrication of aligners via additive manufacturing is not only cost- and time-effective but also opens the door to full digital workflows and individualized treatment planning. However, these systems must first demonstrate mechanical efficacy comparable to industrially produced devices.

In this sense, there has been an exponential growth in interest, both clinical and scientific, in a new material compatible with 3D printing, namely TC-85 DAC resin.

With regard to this recent light-curing resin, a study has been carried out that investigates its peculiar thermo-mechanical and viscoelastic properties [6].

Summarizing the data that emerged, it was found that the thickness of 3D printed aligners fabricated with a DLP printer (Uniz 4K, Uniz, San Diego, CA, USA) with the light-curing resin TC-85 DAC was 12% higher than the value set in the digital design, a value that could affect the biomechanics and efficiency of the aligner.

Furthermore, from the results of the previous study, aligners made with TC-85 DAC resin seem to have a higher flexibility and elastic range than thermoformed PET-G aligners (Easy-Vac Gasket, 3A MEDES, Goyang-si, Republic of Korea).

Tera Harz TC-85 DAC resin has then proved to be geometrically stable at high temperatures and is able to recover its original shape after physical deformation; after being immersed at 80 °C and deformed, the aligner, once returned to a temperature of 37 °C, recovered its original shape over time. In particular, a recovery of more than 50% of the decline within the first minute was observed; the rate of form recovery subsequently gradually decreased. Approximately 90% of the strain was recovered over the next 10 min, and the recovery ratio after 60 min was 96% [6].

In another study, the dimensional accuracy of aligners produced using polyurethane thermoforming sheets (Zendura FLXTM, Zendura Dental, Fremont, CA, USA) on the one hand and Tera Harz TC-85 resin 3D printed with the SprintRay Pro 3D device (SprintRay Inc., Los Angeles, CA, USA) on the other hand was assessed. Printed aligners showed greater accuracy and precision than thermoformed aligners; however, the results of the study may have been affected by some limitations regarding the calculation of the sample size and the method of scanning the cutting surface of aligners [16].

A further study evaluated the cytotoxicity and estrogenicity of aligners printed with TC-85 DAC resin, storing them for 14 days in water at 37 °C. It was found that the eluates released from the resin did not adversely affect the viability of the human gingival fibroblasts that were exposed to them, and no xenoestrogenic activity was observed. Based on these results, it was concluded that the resin tested is biocompatible [17].

The results obtained by this study showed that on average, the Smart Track^®^ aligner with a 0.2 mm expansion per side expresses an average expansion force of about 0.2 N (≈20 g), with a small range of variation (C.V. = 0.0246 N), which suggests that the force exerted by the aligner is constant during all eight hours in which it remains inserted in ElectroForce^®^ (Figure 2).

As for the Smart Track^®^ aligner with a 0.4 mm expansion per side, it emerged that on average, an expansion force of about 0.7 N (≈70 g) is expressed, with a greater range of variation (C.V. = 0.3177 N) than the previous group of aligners. This indicates a variation over time in the force exerted on average by the aligners with 0.4 mm of expansion per side which, as seen in Figure 2, tends to decrease over 8 h.

With regard to aligners made with TC-85 DAC resin, it emerged that those with a planned expansion of 0.2 mm per side express an average contraction force of about 0.0034 N (≈0.3 g), while those with an expansion of 0.4 mm per side express an average expansion force of about 0.014 N (≈1 4 g). Both values indicate a near absence of force expression in Newtons. The ranges of variation in the two groups of TC-85 DAC resin aligners tested are also small (0.0312 N for the former and 0.0239 N for the latter), indicating stability of the aligners in not expressing significant forces during the 8 h (Figure 2 and Table 1).

This raises concerns about their clinical utility in producing expansion movements, as insufficient force application may result in ineffective or unpredictable tooth movement.

Finally, when analyzing the standard deviation of the means of the individual aligner testers, we note that there is considerable variability in the forces expressed by the individual aligners of a group with regard to the Smart Track aligners (Smart Track with 0.2 mm expansion per side: D.S. means ≈ ±0.2 N. Smart Track aligners with 0.4 mm expansion per side: D.S. means ≈ ±0.23 N); this is not noticeable with aligners made with direct TC-85 DAC resin printing (TC-85 DAC resin with 0.2 mm expansion per side: S.D. medium ≈ ±0.02 N. TC-85 DAC resin with 0.4 mm expansion per side: S.D. average ≈ ±0.016 N) (Figure 2 and Table 1).

From this, it follows that, as far as Smart Track aligners are concerned, it is possible to find considerable variability between various aligners that should in theory have an identical conformation to each other. It could be hypothesized that this derives from possible distortions undergone by the material during the aligner fabrication phases.

The hypotheses formulated regarding the outcome of the results showing an ineffectiveness of the aligner made with TC-85 DAC resin in performing this type of displacement, i.e., expansion, focus on possible distortions suffered by the material during the centrifuge and polymerization phases of the resin; the latter phase in particular is operator-dependent and requires manual phases as well as various manipulations in order to remove excess resin and the physical supports that allow the printing of the aligner itself.

Although not directly addressed in this study, practical recommendations arise from the theoretical implications of our results and illustrate their potential relevance across various engineering applications. The cleaning procedure must be carried out before final curing, but at a stage in which, in theory, the aligner should already be stable, as stated in the indications given by the manufacturers, so that it can be handled without the risk of deformation.

Inconsistent post-processing protocols, especially manual handling, could compromise the mechanical integrity of printed aligners. Standardized and validated workflows are crucial to ensure consistent performance in a clinical setting.

In the present study, a posterior crossbar was also added during the design phase, which would act as a transversal support to the aligner during all the manual procedures described above and eliminated only at the end of the final polymerization of the product in order to minimize these occurrences; however, judging by the results obtained, this precaution may not have been sufficient or other variables that alter the final product may be involved.

Ideas for future research could be to see if the forces involved could approach those expressed by Smart Track by modifying the geometry of the aligners, for example, by increasing the extension of the flanges, covering the palatine vault with the aligner for the upper arch, or thickening the aligner in strategic points (such as the lingual portion of the same for the lower arch) in order to simulate with the resin a sort of palatine bar or lingual arch activated in expansion according to the design of the case.

It is important to mention that 0.75 mm thickness was used for the 3D printed aligners in order to obtain a comparable thickness to Smart Track aligners. Currently, in clinical practice, 3D printed aligners using TC-85 DAC resin are fabricated with a thickness of 0.5 mm [18]; different results may occur when using different thicknesses.

In light of the results obtained, this study is certainly worthy of further investigation, increasing the sample size or comparing other types of material available today for the creation of 3D printed orthodontic aligners.

In the end, this study was performed in 2023 and the new resin TA-28 was not available; different results could be highlighted with further analysis with this new material [19].

Another limitation of the study is that the data are derived from one single patient record that was used as a starting point to fabricate the aligners.

Although a number of recent reviews and book chapters have addressed the topic of 3D printable materials for intraoral orthodontic use, the current body of evidence remains limited in both scope and depth. Further research is warranted to fully explore the clinical potential of this technology, which shows promising features deserving more comprehensive investigation [20,21,22,23,24,25].

## 5. Conclusions

In conclusion, we can say that Smart Track^®^ aligners express a quantitatively measurable force in Newtons during the programmed movements to obtain a posterior expansion of the dental arches; this strength decreases slightly over time (as observed in aligners with an expansion of 0.4 mm per side). It can also be concluded that there is variability between multiple types of Smart Track aligners that are programmed to be identical to each other.

TC-85 DAC resin expresses forces close to 0 during the realization of the programmed movements to obtain a posterior expansion of the dental arches; this result does not change if the amount of programmed expansion is modified, remaining the same if an amount of 0.2 mm or 0.4 mm of expansion is programmed per side. 

Further studies are needed to deeply investigate the topic.

## Figures and Tables

**Table 1:** Average values of registered strength.

Device	Average Strength (N)	Standard Deviation (N)	Field of Variation in The Average (N)
TC-85 DAC exp 0.2 mm	−0.0034 N	±0.0202 N	0.0312
TC-85 DAC exp 0.4 mm	+0.0141 N	±0.0167 N	0.0239
Smart Track exp 0.2 mm	+0.2162 N	±0.2001 N	0.0246
Smart Track exp 0.4 mm	+0.7159 N	±0.2372 N	0.3177

## Data Availability

The full anonymized dataset of the study was opened and available from the corresponding author when it is required.
